# Editorial: Frontiers in Synaptic Plasticity: Dendritic Spines, Circuitries and Behavior

**DOI:** 10.3389/fpsyt.2016.00112

**Published:** 2016-06-20

**Authors:** Alberto A. Rasia-Filho, Rochelle S. Cohen, Oliver von Bohlen und Halbach

**Affiliations:** ^1^Department of Basic Sciences/Physiology, Federal University of Health Sciences, Porto Alegre, Brazil; ^2^Department of Anatomy and Cell Biology, University of Illinois at Chicago, Chicago, IL, USA; ^3^Institute of Anatomy and Cell Biology, Universitätsmedizin Greifswald, Greifswald, Germany

**Keywords:** synaptic function, synaptic transmission, synaptic remodeling, dendritic spine function, neural circuits and behavior, sexual dimorphism, neuroimmune interactions, psychiatric disorders

**The Editorial on the Research Topic**

**Frontiers in Synaptic Plasticity: Dendritic Spines, Circuitries and Behavior**

More than a century ago, in 1906, the Nobel Prize in Physiology or Medicine was awarded to Camillo Golgi and Santiago Ramón y Cajal “in recognition of their work on the structure of the nervous system.” Using the Golgi technique, Cajal discovered and described dendritic spines, which, since then, have received considerable attention. Dendritic spines are the major targets of excitatory synapses within the brain. Their disparate morphologies appear to reflect cellular processes involved in neuronal and synaptic plasticity. Dendritic spines reach high levels of complexity in humans (1). Neuronal and synaptic plasticity are manifested by changes in structure (e.g., dendritic spine shape, size, density, and connectivity) and activity (e.g., long-term potentiation) leading to dynamic changes in circuitries for neuronal processing. Furthermore, some of these changes in the brain can translate into altered behavior and even can contribute to psychiatric disorders. Animal models have been key to the study of affective and social behaviors, as well as neurological and psychiatric disorders. They provide insight into mechanisms underlying basic to complex neural functions and disturbances in behavior. However, there is a paucity of compilations correlating alterations in synaptic structure with various physiological and behavioral paradigms. This Research Topic is a forum for the exchange of data and novel hypotheses about synaptic and brain plasticity. It comprises 10 articles with 3 original research articles, 3 reviews, 2 hypothesis and theory papers, 1 opinion, and 1 general commentary elaborated by 39 authors from various countries. These contributions present state-of-the art approaches to the study of dendritic spines, circuitries, and behavior from animal models, including rodents and primates, to humans. The research strategies used range from classic techniques to cutting-edge technologies, including imaging techniques, electrophysiology, and experimental-based hypothetical approaches.

Tønnesen and Nägerl provide up-to-date STED microscopy data on structure and function relationships of dendritic spines. These data include the spine head volume and local postsynaptic density associated with the neck diameter and its variable resistance. In conjunction, they modulate the spine electrical compartmentalization or the influence on dendritic voltage and synaptic plasticity. These data are crucial in evaluating the impact of spine geometry on neuronal function and dynamic synaptic processing and enduring changes in neural circuits.

Hansberg-Pastor et al. describe the broad and complex actions of estradiol and progesterone on the regulation of protein components of the cytoskeleton of neurons and astrocytes that ultimately affect cellular morphology, function, and connections, including dendritic spine growth. These properties correlate with region-specific features in the brain of females. This modulation begins early in development and persists along the life span, notably during the estrous cycle, suggesting a continuous plastic transformation of dendritic spines, synapses, and neural networks.

Bittencourt et al. demonstrate that synapses and circuitries can undergo post-lesion reorganization, as is the case for mossy fiber sprouting and its debatable relationship with epileptogenesis. Here, ultrastructural data reveal the number and type of asymmetric contacts involving spine and shaft synapses and the likely restorative connectivity of the dentate gyrus molecular layer of rats with induced chronic seizures.

Vargas-Barroso et al. report cytological, whole-cell patch-clamp electrophysiological and connectional data indicating an anatomical and functional interaction between the accessory and the main olfactory bulb in rats. These findings are relevant for the animal’s perception of complex chemosensory clues from the environment and the neural circuits implicated in the display of various social behaviors.

Calcagnotto addresses the role of interneurons in synaptic plasticity and the ways in which cellular replacement approaches can rescue defects in local circuit activity and synaptic plasticity. Strategies that alter interneuronal networks, including those that control inhibitory interneurons and the use of precursor cell grafts, may have the potential to restore synaptic plasticity and brain oscillations.

Zimmermann-Peruzatto et al. provide a comprehensive review of the relationship between vasopressin receptors and specific brain circuits. Alterations in vasopressinergic pathways may lead to changes in synaptic plasticity and parental and sexual behaviors.

de Sousa et al. link important data about the secretion and modulatory actions of hormones to developmental ages in young primates. They show that sex differences, cortisol levels, acute or chronic social isolation, and coping strategies are important for the development of neural circuitries and learning in male and female monkeys, an approach that can serve as a model for the study of emotional and behavioral disorders.

Gottfried et al. propose that neuroimmune responses are central to translating the effect of environmental risk factors and genetic and epigenetic changes to deficits in brain function and behavior in autism spectrum disorder (ASD). They present an immunological sequence of events leading to neuroinflammation, neuronal-gial responses, and brain connectivity dysfunction that may be involved in ASD pathogenesis.

Siniscalo highlights the impact of aberrations in neuroimmune responses in ASD, as proposed by Gottfried et al., citing the role of pro-inflammatory cytokines in disruption of the immunological interface between the peripheral immune system and central nervous system, leading to deleterious neuronal and behavioral consequences.

Pandey’s group (Kyzar et al.) provides evidence for the effects of disturbances in epigenetic programming during adolescence due to repeated exposure to binge levels of alcohol. Alcohol exposure during adolescence leads to alterations in epigenetic, neurotrophic, and neuroimmune pathways in the brain, manifested by widespread and persistent changes in synaptic remodeling and neurogenesis in strategic brain areas. Rodent and human data link alcohol exposure, impaired dendritic spines, and neural circuitry to long-lasting behavioral consequences in the adult.

We thank the reviewers for their valuable contribution and the outstanding support and efforts of Dr. Raina Robeva, Specialty Chief Editor, Frontiers in Systems Biology; Dr. Kathleen Dave for editing the commentary; the members of the Editorial Office of Frontiers in Psychiatry and Frontiers in Neuroscience, Mr. Dimitri Christodoulou, Mrs. Sara Fahmy, Mrs. Andrea Polonioli, and the Journal Manager Dr. Jessica Kandlbauer. In memoriam of Dr. Craig H. Kinsley (University of Richmond, USA) who initially contributed the abstract entitled “Maternal neurons generate maternal behavior.”

## Author Contributions

AARF, RSC, and OvBuH are co-editors of this research topic.

## Conflict of Interest Statement

The authors declare that the research was conducted in the absence of any commercial or financial relationships that could be construed as a potential conflict of interest.
